# Innovative Strategy for Aroma Stabilization Using Green Solvents: Supercritical CO_2_ Extracts of *Satureja montana* Dispersed in Deep Eutectic Solvents

**DOI:** 10.3390/biom13071126

**Published:** 2023-07-14

**Authors:** Jelena Vladić, Strahinja Kovačević, Krunoslav Aladić, Stela Jokić, Sanja Radman, Sanja Podunavac-Kuzmanović, Ana Rita C. Duarte, Igor Jerković

**Affiliations:** 1 LAQV-REQUIMTE, Department of Chemistry, NOVA School of Science and Technology, NOVA University Lisbon, 2829-516 Caparica, Portugal; 2 Faculty of Technology Novi Sad, University of Novi Sad, 21000 Novi Sad, Serbia; 3 Faculty of Food Technology Osijek, University of Josip Juraj Strossmayer of Osijek, 31000 Osijek, Croatiasjokic@ptfos.hr (S.J.); 4 Faculty of Chemistry and Technology, University of Split, 21000 Split, Croatia; sanja.radman@ktf-split.hr

**Keywords:** volatile compounds, *Satureja montana*, deep eutectic solvent, aromas, stabilization, supercritical carbon dioxide

## Abstract

The aim of this work was to establish the potential of natural deep eutectic solvents (NADES) for the stabilization of aroma volatile organic compounds from a natural source. *Satureja montana* was used as a source of volatile components, as it is rich in terpenes of great commercial and biological importance, such as carvacrol, thymol, and thymoquinone, among others. Supercritical CO_2_ was used to extract the lipophilic fraction of *S. montana*, which was further directly dispersed in NADES. The stabilizing capacity of seven different NADES based on betaine and glycerol was analyzed. The stability of the components in NADES was monitored by analyzing the headspace profile during 6 months of storage at room temperature. The changes in the headspace profile over time were analyzed by using different statistical and chemometric tools and the Wilcoxon matched pair test. It was determined that alterations over time occurred such as degradation and oxidation, and they were the most prominent in the control. In addition, the indicator of decreased stability of the control was the formation of the new compounds that could compromise the quality of the product. In the stabilized NADES samples, the changes were significantly less prominent, indicating that the NADES had a stabilizing effect on the volatile compounds. According to Wilcoxon matched pair test, the most efficient stability was achieved by using betaine/ethylene glycol, glycerol/glucose, and betaine/sorbitol/water. Therefore, by applying two green solvents, a sustainable approach for obtaining pure and high-quality *S. montana* extracts with extended stability at room temperature was established.

## 1. Introduction

Volatile organic aroma compounds represent important constituents in various fields because they possess sensory and numerous biologically important properties. These components are incorporated into products intended for the medical treatment of humans and animals, cosmetic and food products, beverages, fragrances and perfumes, products for care and cleaning, insecticides, repellents, etc. Several markets drive the demand of volatile organic aroma compounds, including the food and beverage (35%), fragrances, cosmetics and aromatherapy (29%), household (16%), and pharmaceutical (15%) markets [1,2]. Numerous industries use synthetic compounds, as the process of obtaining them from natural sources can be expensive and irrational. However, the connection of synthetic components with negative consequences on health and the environment, together with the growth of green consumerism, represent the reason for the growing global demand for natural aroma compounds, their implementation in products, and the cessation and/or reduction in the use of their synthetic counterparts [1].

Therefore, the growing demand and wide applicability of aroma compounds pose new challenges and require new trends in production. Conventional production procedures, such as hydrodistillation and extraction with organic solvents that are harmful to human health and the environment, cannot ensure the rational use of natural raw materials and a high-quality and safe product in adequate quantities in an environmentally benign production process. Additionally, aroma compounds are characterized by instabilities, easy reactivities, volatilities, and tendencies to degradation; therefore, their application is difficult [3]. Apart from organoleptic alterations and impairment of the compounds’ biological properties, is it also possible to compromise the consumers’ well-being, for example, by manifesting skin-sensitizing effects [3]. In order to prevent the deterioration of the quality and biological properties, it is necessary to ensure the stability of aroma compounds during the process of isolation, processing, and storage.

Furthermore, addressing the mentioned challenges is in accordance with the sustainable development agenda [4], as well as with the objectives of green chemistry, as it involves the creation of strategies that include the efficient use of natural renewable resources, attainment of long-term stable aroma compounds, elimination of the use of harmful solvents, waste reduction, and greater process efficiency.

For aroma preservation, the most industrially relevant and represented technique is spray drying. However, this technology implies high process temperatures, which can be a huge disadvantage for thermosensitive components. Also, numerous techniques have been developed for the preservation of aroma volatile compounds such as fluidized bed coating, freeze-drying, spray cooling and spray chilling, extrusion, co-crystallization, simple or complex coacervation, inclusion complexation, ionic gelation, the formation of liposomes, etc. [5,6]. In addition to ensuring high quality and preventing the loss of volatile compounds during obtaining and processing the product, a very important aspect is the simplicity and economy of the process. All preservation techniques represent additional processes after aroma attainment and require additional equipment. Therefore, the development of simple and economic procedures for obtaining and preserving aroma volatile compounds is necessary.

Supercritical CO_2_ is a green solvent that has been introduced into industrial processes as a sustainable alternative to conventional hazardous organic solvents. Supercritical CO_2_ is suitable for the efficient recovery of lipophilic aroma components from natural materials and enables obtaining extracts of a good safety, quality, and purity profile. When CO_2_ is in a supercritical state (critical temperature 31.06 °C and critical pressure 7.39 MPa), by changing the process parameters, it is possible to adjust the thermophysical properties of the solvent and the characteristics of the product. In addition to being safe for work, cheap, and available, its advantage is that it is easily removed from the product and reused while the product does not need to be purified. Moreover, this results in reduced energy consumption, lower costs, and increased sustainability of the process. Additionally, degradation is avoided because the process is carried out at low temperatures [7,8].

The new generation of alternative solvents, natural deep eutectic solvents (NADES), comprise mixtures of components of natural origins that form intermolecular interactions when mixed in an appropriate molar ratio. The bonds they form are mainly through hydrogen bonding, which results in a significant depression of the melting point of the mixture and the formation of a liquid mixture. What distinguishes them is easy preparation with 100% atom economy, thermal and chemical stability, low-cost, non-flammability, biodegradability, and absence of or low toxicity. NADES mixtures are tailor-made solvents; hence, it is possible to tune their physico-chemical properties, making them versatile solvents in different fields of applications [9,10]. There are literature data that indicate the stabilizing properties of NADES for different types of components [11]. However, the potential of NADES for stabilizing aroma volatile organic compounds has not been investigated previously.

Therefore, the goal of this study was to establish a novel green approach of using environmentally friendly solvents to enable the attainment of aroma volatile compounds and maintain their stability at room temperature. *Satureja montana* L. (*Lamiaceae*) was selected for research, as it represents a rich source of volatile aroma compounds such as carvacrol, thymol, and thymoquinone, which are highly important and have applications in various fields. Due to its strong affinity for volatile compounds, supercritical CO_2_ was used for the isolation of volatile compounds and obtaining clean and safe extracts, which were further stabilized using NADES. The stabilizing capacity of NADES mixtures was evaluated by monitoring the chromatographic changes in the volatile profile of the CO_2_ extracts dispersed in different NADES during storage for 6 months. For data analysis and the assessment of the stability of the samples during time, the Wilcoxon matched pair test and different chemometric methods (hierarchical cluster analysis, principal component analysis, and a ranking approach—sum of ranking differences) were used with the aim of establishing adequacy of different NADES for the stabilization of *S. montana* volatile aroma compounds.

## 2. Materials and Methods

### 2.1. Materials and Chemicals

*Satureja montana* L. aerial parts were obtained from the Institute for Medicinal Plants Research “Dr. Josif Pancic”, Belgrade, Serbia. The mean particle size (0.32 ± 0.05 mm) of the material was determined using vibration sieve sets (CISA, Cedaceria, Barcelona, Spain). Moisture content of the plant material (9.26%) was determined using a moisture analyzer DAB (Kern, Ballingen, Germany).

Betaine (≥99%), D-glucose monohydrate (≥97.5%), D-sorbitol (≥98%), and sucrose (≥99.5%) were purchased from Sigma-Aldrich (St. Louis, MI, USA); glycerol (99.5%) was purchased from Scharlau (Barcelona, Spain); ethylene glycol (≥99.5%) was obtained from Carlo Erba (Val-de-Reuil, France); L-proline (≥ 99%) was purchased from Alfa Aesar (Haverhill, MA, USA).

### 2.2. Preparation and Characterization of NADES

NADES mixtures were prepared by mixing components in an adequate molar ratio and then heating (40 °C) and stirring until a clear liquid was formed. In this work, different NADES based on betaine and glycerol were used. The NADES that were applied, abbreviation, and molar ratio are listed in Table 1.

### 2.3. Determination of Viscosity

The viscosity of the different NADES (Bet/Sor/W, Bet/Suc/Pro/W, Gly/Glu/Sorb/W, Pro/Gly/Sorb/W) was determined using a MCR102 Modular Compact Rheometer (Anton Parr) fitted with a parallel plate geometry with 50 mm of diameter (PP50, Anton Parr) and 1 mm of gap. Measurements were conducted in triplicates. Viscosity of Gly/Glu, Bet/EG, and Bet/Gly/Suc/W was determined previously [12,13].

### 2.4. Preparation of Samples

#### Supercritical Carbon Dioxide Extraction and Dispersion in NADES

Supercritical CO_2_ extraction was carried out using a high-pressure extraction system (HPEP, NOVA-Swiss, Effretikon, Switzerland) with the following main specifications: gas cylinder with CO_2_, diaphragm-type compressor (with a pressure range up to 1000 bar), extractor vessel with heating jacket (internal volume 200 mL, maximum operating pressure 700 bar), separator with cooling jacket (internal volume 200 mL and maximum operating pressure 250 bar), pressure control valve, temperature regulation system, and regulation valves. The extractions were performed under the following conditions: pressure 300 bar, temperature 40 °C, CO_2_ flow 0.194 kg/h, and extraction time 4.5 h. The parameters of extraction were selected based on the previously published work [14]. Obtained extracts were placed in glass bottles and stored at 4 °C prior to further analysis. Each extraction was performed in triplicate.

After 4.5 h of extraction, to separate the CO_2_ and extract, depressurization was performed, and the extract was directly dispersed into the prepared NADES mixture. The mixture of CO_2_ extract and NADES was homogenized by mixing in a vortex for 1 min. The extract: NADES ratio was 0.05 g ± 15% extract: 1 mL NADES. This ratio was chosen because it enabled easy homogenization of the mixture. The control and CO_2_ extracts that dispersed into NADES (CO_2_-NADES systems) were analyzed at the beginning (month 0), after 3 months, and after 6 months of storage in transparent containers at room temperature in the dark.

### 2.5. Headspace Solid-Phase Microextraction (HS-SPME)

Volatiles in the headspace were extracted using a solid phase microextraction (SPME) fiber installed on the PAL Auto Sampler System (PAL RSI 85, CTC Analytics AG, Schlieren, Switzerland). The fiber was covered with a layer of divinylbenzene/carbon wide range/polydimethylsiloxane (DVB/Carbon WR/PDMS) and was purchased from Agilent Technologies (Palo Alto, Santa Clara, CA, USA). Before the analysis, the fiber was conditioned according to the manufacturer’s instructions. The sample (1.5 g) was placed in a 15 mL glass vial and hermetically sealed with PTFE/silicone septum. The sample was equilibrated at 60 °C for 15 min, and extraction was performed for 45 min, followed by 6 min thermal desorption through the 250 °C inlet and directly onto the GC column.

### 2.6. Gas Chromatography with Mass Spectrometry Analysis (GC-MS)

An Agilent 8890 gas chromatograph (Agilent Technologies, Palo Alto, CA, USA) coupled to a mass spectrometer (series 5977E, Agilent Technologies, Palo Alto, CA, USA) was used for the analysis. The analyzed components were separated in a HP-5MS capillary column (30 m × 0.25 mm, 0.25 μm, Agilent Technologies, Palo Alto, CA, USA). The GC conditions were described previously [15].

Qualitative identifications of the compounds were performed using the Wiley 9 (Wiley, New York, NY, USA) and NIST 17 (National Institute of Standards and Technology, Gaithersburg, MD, USA) mass spectral libraries, as well as the literature data of retention indexes calculated with C_9_–C_25_ alkanes. The HS-SPME/GC-MS analysis of each sample was performed in three replicates, and the results (relative percentages) were expressed as mean data.

### 2.7. Chemometric Methods

The chemometric analysis was performed on natural data since all the variables were on the same scale. HCA was carried out using the NCSS 2023 program [16]. The clustering of the samples and variables was presented on clustered heat maps (double dendrogram) to clearly show based on which variables the samples were grouped in the clusters. The clustering methods were Ward’s variance, and the distance method was based on Euclidean distances.

PCA was performed by using Statistica v. 14.0.0.15 program [17]. The analysis was based on correlations. The relevant number of PCs was estimated based on Eigenvalues higher than 1. The results of the PCA were presented in the form of scores and loadings plots that represented the distribution of the samples in the space of the considered variables.

SRD analysis was performed by using a program created in Microsoft Excel software, created by Héberger and Kollár-Hunek [18,19]. The method was based on the calculation of the sum of ranking differences between the objects (samples, models, etc.) and a reference ranking. In the present study, the reference ranking was the row average values (consensus ranking). The SRD modeling was validated by Comparison of Ranks by Random Numbers (CRRN) approach and 7-fold cross-validation procedure [19].

## 3. Results and Discussion

Instability, volatility, reactivity, and inclination towards degradation are responsible for significant changes in aroma volatile components, which altogether has significant economic and health consequences. These deteriorations are particularly expressed at room temperature [3,20]. Therefore, storage at room temperature was chosen for stability testing. By ensuring the stability of aroma volatile compounds at room temperature, the application of these components is facilitated, the storage and transportation processes of such products are simplified, and health risks are reduced.

Easily available and low-cost constituents were selected for the design of NADES: glucose, sucrose, glycerol, sorbitol, proline, betaine, and ethylene glycol. The viscosity of NADES could represent an obstacle to applying NADES; however, it could potentially have a positive effect on the stabilizing capacity of NADES because it slows down the movement of molecules [21]. Therefore, NADES with wide viscosity ranges were used in this work (Table 1).

At the beginning (month 0), the headspace composition of *S. montana* extracts dispersed in different NADES (CO_2_-NADES systems) was established, followed by monitoring of the changes in the headspace profiles of the CO_2_-NADES systems after 3 and 6 months of storage at room temperature. CO_2_ extract was used as a control, and its volatile profile was monitored in the same way.

The *S. montana* samples were found to be complex mixtures of components belonging to different chemical groups, such as hydrocarbons, alcohols, aldehydes, esters, ethers, ketones, phenols, and oxides. A total of 69 components were identified in the samples, which made up 83.06–98.97% of the headspace profile of the samples (Table 2, Table 3 and Table 4). Oxygenated terpenes were predominant in the samples and accounted for 53.99–86.05% of the volatile profile, followed by sesquiterpene hydrocarbons 3.29–28.03%, monoterpene hydrocarbons 1.01–9.38%, oxygenated sesquiterpenes 0.17–13.27%, and non-terpenes 0.40–3.97%. Table 2, Table 3 and Table 4 show the headspace composition of the control and CO_2_-NADES systems at the start (month 0), after 3 months, and after 6 months of storage.

Among the most dominant components in the samples were the isomeric monoterpene phenols carvacrol and thymol (6.38 ± 0.97–31.44 ± 3.21% and 5.65 ± 0.36–23.48 ± 1.77%, respectively). These two components are of exceptional commercial importance and have a wide spectrum of applications in other fields such as pharmacy, pharmacy, medicine, dentistry, cosmetics, food, veterinary, and agrochemistry [22]. According to the literature, *S. montana* lipophilic extracts and essential oils mainly belong to phenolic chemotypes with dominant carvacrol and/or thymol. Therefore, the distribution of components established in this work was in accordance with the literature [23,24].

**Figure 1:**
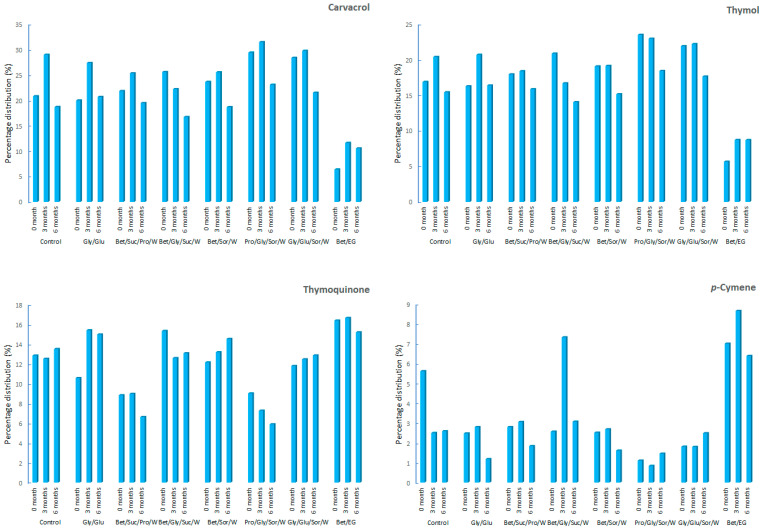
Percentage distribution of the most significant compounds (thymol, carvacrol, thymoquinone, and *p*-cymene).

Monitoring of the headspace profile (during 6 months) revealed changes in the chemical profile of the samples as a function of storage time. In the Bet/Gly/Suc/W sample, a constant decrease in thymol (from 20.84 ± 2.68 to 14.02 ± 3.14%) and carvacrol (from 25.56 ± 4.22 to 16.70 ± 2.50%) was observed during storage. Moreover, in the Bet/EG sample, thymol and carvacrol remained almost unchanged in percentage after the initial increase at 3 months, even after 6 months (Figure 1). In other samples, carvacrol had an increasing trend (after 3 months) and then a decrease after 6 months. Thymol in the control and Gly/Glu exhibited the same trend—an increase (after 3 months) followed by a decrease. While, in the other samples (Bet/Suc/Pro/W, Bet/Sor/W, Pro/Gly/Sor/W, and Gly/Glu/Sor/W), thymol had small oscillations in abundance after 3 months, followed by a decrease (after 6 months), possibly indicating the preserved stability of thymol in the first three months in these samples.

The initial presence of carvacrol and thymol in the Bet/EG sample was significantly lower (6.38 and 5.65 ± 0.36%, respectively), compared to the remaining samples (thymol 16.25 ± 2.57–23.48 ± 1.77% and carvacrol 19.96 ± 3.04–29.37 ± 3.02%). Also, the distribution of other less abundant components in this sample was different from the remaining CO_2_-NADES systems and control. This difference could be explained by the different batches and non-uniformity of the material that was the commercial sample. Therefore, the monitoring of all samples from the beginning (month 0) to 6 months was of great importance.

Another important constituent that bettered the quality of the *S. montana* extract was thymoquinone. This component is classified as a promising candidate for drug development because it exhibits numerous pharmacological activities, high therapeutic indexes, favorable pharmacokinetics, and good safety and toxicity profiles [25]. Determined thymoquinone oscillations in the samples over time could potentially be a consequence of changes in the abundance of thymol and carvacrol because thymoquinone can be formed by subsequent oxidation of thymol and carvacrol. However, it was not possible to clearly establish these processes because most likely other components were included in the sequential oxidative reactions. For example, the changes in the presence of thymol, carvacrol, and thymoquinone could also be caused by changes in *p*-cymene, the precursor of thymol and carvarol. Furthermore, changes in *p*-cymene content could be preceded by other changes, such as the cyclization of myrcene to *α*- and *γ*-terpinenes and limonene, which may be further converted to *p*-cymene by rearrangement, hydrogenation, or dehydrogenation [26].

*p*-Cymene was dominant (0.84 ± 0.17–8.65 ± 2.58%) in the group of monoterpene hydrocarbons. In addition to having significant pharmacological properties [27], its presence in samples is essential because it is considered responsible for the more effective manifestation of the antimicrobial action of carvacrol. Namely, due to its hydrophobicity, it has a high preference for cytoplasmic membranes, allowing carvacrol to easily enter into the cell [28]. *p*-Cymene as a monoterpene hydrocarbon is more susceptible to changes compared to its oxidation derivatives. In the control, the stability of *p*-cymene was low compared to the CO_2_-NADES samples; so, its abundance in the control was reduced more than twice after 3 months, which coincided with the initial growth of carvacrol and thymol in the control. In the Bet/Gly/Suc/W sample, after 3 months, an increase in *p*-cymene of 2.8 times was recorded and, after that, a decrease to a percentage close to the initial one. In the other samples, *p*-cymene had significantly less oscillation over time, so its content was close to the initial one after 3 months. The highest abundance of *p*-cymene was in Bet-EG, where its percentage after 6 months (6.38%) was close to that at the start (7%).

Furthermore, a reduction in carvacrol methylether was observed in the samples, which may have been due to its degradation to carvacrol and *p*-cymene [29]. Linalool increased in all samples after 3 months, and it increased even after 6 months in most of them, with the exception of Bet/Gly/Suc/W and Bet/Sor/W, where it stagnated after the initial growth. Growth of linalool has been reported during storage of lemon-flavored hard tea at room temperature [30]. According to He et al. [30], the increase in linalool can occur due to autoxidation of myrcene [31], which, in the samples, was detected only at the beginning and also in Bet/EG in traces after 3 months. The increase in a minor compound α-terpineol, which can be formed by oxidation of limonene, was also detected. The changes in the monoterpene alcohols terpinene-4-ol and borneol could also be attributed to the degradation reaction of hydrocarbons.

The group of oxygenated sesquiterpenes showed an increase in the samples, corresponding to a decrease in sesquiterpene hydrocarbons over time. In the group of oxygenated sesquiterpenes, a total of three components were identified, with an increasing trend over time in all samples compared to the start, which was caused by the transformations of sesquiterpene hydrocarbons. The appearance of caryophyllene oxide after 3 months and an increase up to 6 months could be attributed to the oxidation of *β*-caryophyllene, which decreased over time. An increase in caryophyllene oxide was reported in the literature as one of the characteristic indicators of the aging of essential oil and lipophilic products [3]. The identification of viridiflorol in the samples after 6 months was in accordance with the decrease and disappearance of the corresponding sesquiterpene aromadendrene hydrocarbon ledene (viridiflorene). Also, apart from viridiflorol, another aromadendron derivative, alcohol spathulenol, was identified.

In the control, a decrease in monoterpene hydrocarbons was observed approximately 3 times after 3 months. This trend in hydrocarbons in the control was in accordance with the literature’s results, where a decreasing trend in low boiling hydrocarbons at room temperature storage was reported. Most likely, evaporation, oxidation, and other unwanted changes in the product constituents during the storage period were responsible for such changes [20]. On the other hand, in the CO_2_-NADES systems, the changes in the presence of hydrocarbons were significantly milder, mostly with a decreasing trend, potentially indicating that NADES improved the stability of these components compared to the control. The only CO_2_-NADES system in which a more pronounced oscillation (increase) was determined in the presence of this group of components was Bet/Gly/Suc/W.

Unstable constituents, such as hydrocarbons, are susceptible to chemical transformations due to their interactions with other components; hence, their stabilization is important for the stability of the entire product. As a result of the oxidation of the components, changes in the smell, taste, and texture of the product may occur. In addition, oxidation and other degradation reactions can lead to the formation of low molecular weight volatile components that exhibit undesirable effects on human health and lead to undesirable odors and flavors [32,33].

In the group of non-terpenes, several components were identified that were not initially present in the samples. The largest number of newly formed components, as well as the largest increase in the presence of non-terpene compounds, was observed in the control. In control after 6 months *E*,*E*-hepta-2,4-dienal was identified. This is a polyunsaturated aldehyde that can be developed due to a series of enzymatic transformations of mainly free polyunsaturated fatty acids. It is usually detected in stored oil samples with reduced stability and points to the oxidative changes related with lipid food spoilage and leads to the rancid defect [34]. Also, after 6 months, γ-butyrolactone, a decomposition product of tetrahydrofuran, was identified in the control, which was found to be acutely toxic at low concentrations in larval zebrafish [35]. Acetic acid was detected only in the control samples and over time its presence increased (up to 1.45%). This growth of acetic acid could indicate the reduced stability of the control. Huang et al. [36] stated that acetic acid can appear as a by-product of furan formation. Furthermore, the presence of acetic acid was reported as a consequence of undesirable processes during storage of honey and was accompanied by the development of an unpleasant and sour taste [37]. 4-Methylbenzaldehyde, the benzaldehyde derivative, was detected in all samples after 6 months, except for Gly-Glu and Bet/EG. Benzaldehydes are used as food preservatives; however, it has recently been established that prolonged exposure to benzaldehydes is associated with cancer and disorders of the liver, kidney, nervous, and reproductive systems [38].

### 3.1. Hierarchical Cluster Analysis and Principal Component Analysis

Statistical and chemometric methods were applied for further analysis and interpretation of the obtained results, which can be a useful tool in the analysis of large data sets [39,40,41]. In this work, the pattern recognition methods HCA and PCA, a ranking approach SRD, and Wilcoxon matched pair test were applied.

**Figure 2:**
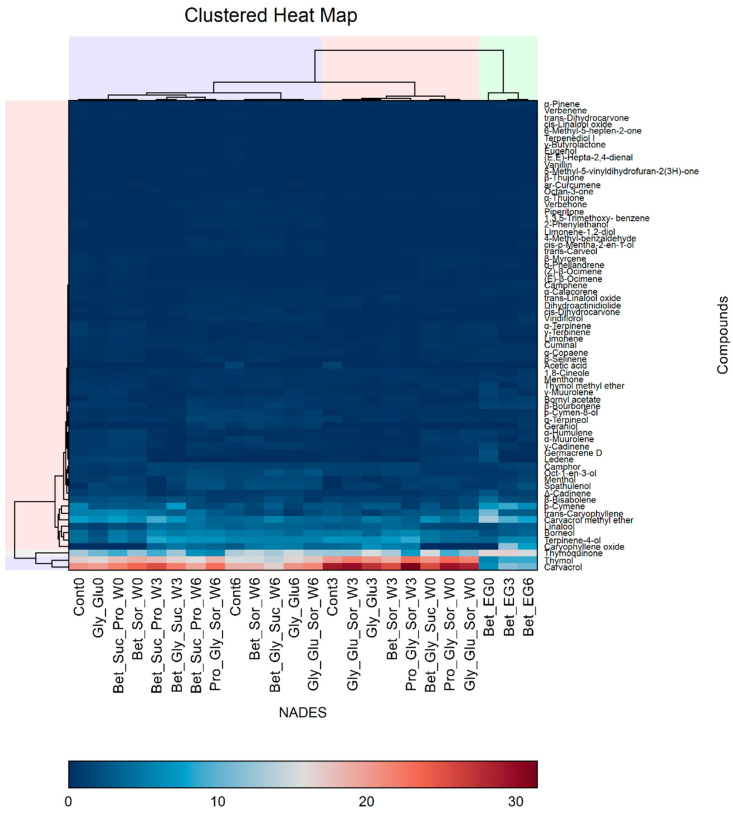
Clustered heat map (double dendrogram) of the control and CO_2_-NADES systems and the compounds detected in the extracts. The blue–red scale presents the percentage distribution of compounds in each sample.

The HCA resulted in two double dendrograms. The dendrogram that represents the clustering of the control and CO_2_-NADES systems based on the percentage of each detected compound is given in Figure 2. The presented vertical dendrogram indicates the following findings: (1) the most abundant compounds in almost all extracts were carvacrol and thymol (placed in the purple vertical cluster), except in the Bet/EG extracts, in which thymoquinone was the most abundant compound; (2) the significant presence of borneol, terpinene-4-ol, caryophyllene oxide, linalool, carvacrol methyl ether, *trans*-caryophyllene, and *p*-cymene was observable in the majority of the extracts (placed in the separate vertical sub-cluster of the red-colored cluster); (3) the majority of the compounds were minor compounds, located in another sub-cluster of the vertical red cluster.

The horizontal dendrogram in Figure 2 shows the grouping of the CO_2_-NADES systems and control samples. It indicates that all three control samples were placed in different clusters and sub-clusters, suggesting that they were potentially different and, therefore, not very stable over time. Bet/EG samples were placed in a separate cluster (green horizontal cluster), which indicated that they were distinctive from the other CO_2_ -NADES systems, as previously explained. The main reason for their separation into different clusters was the content of thymoquinone, which was higher than in other CO_2_-NADES systems, and the significantly lower abundance of thymol and carvacrol in comparison with other extracts. The distribution of other NADES in the purple and red horizontal clusters showed that some samples monitored at the start and after 3 months were placed in the same cluster (e.g., Gly/Glu/Sor/W0 and Gly/Glu/Sor/W3, as well as Pro/Gly/Sor/W0 and Pro/Gly/Sor/W3, which belong to the red horizontal cluster), as well as some samples monitored after 3 and 6 months (Bet/Gly/Suc/W3 and Bet/Gly/Suc/W6). The main observations were scattering of the control samples in the dendrogram and significant separation of Bet/EG samples from other samples but in the same cluster, indicating that the Bet/EG samples had not suffered significant changes over time.

**Figure 3:**
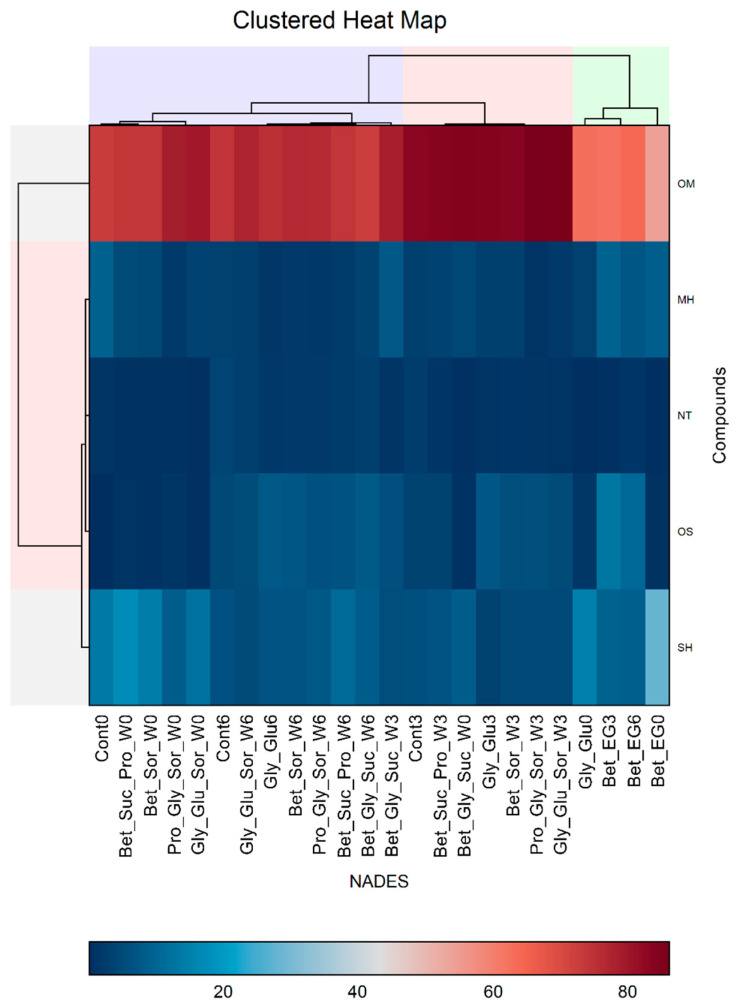
Clustered heat map (double dendrogram) of the NADES and the group of compounds (OM—Oxygenated Monoterpenes, MH—Monoterpene Hydrocarbons, NT—Non-Terpenes, OS—Oxygenated Sesquiterpenes, SH—Sesquiterpene Hydrocarbons) detected in the extracts. The blue–red scale presents the percentage distribution of a group of compounds in the samples.

Another aspect of HCA of CO_2_-NADES systems and control is presented in Figure 3, where the clustered heat map shows the clustering of the samples in regard to the groups of compounds (oxygenated monoterpenes (OM), monoterpene hydrocarbons (MH), non-terpenes (NT), oxygenated sesquiterpenes (OS), and sesquiterpene hydrocarbons (SH)).

The vertical dendrogram indicates the significant separation of the OM compounds considering their highest contents (it can be considered as an outlier since it is placed outside the main cluster), whereas the other groups of compounds (MH, NT, OS, and SH) belonged to the same cluster considering their abundance, which was significantly lower in comparison to the OM compounds. The clustering of the CO_2_-NADES and control samples is presented in the horizontal dendrogram, which indicates the scattering of the control samples in different clusters and sub-clusters. Moreover, it also demonstrates the grouping of Bet/EG samples in a similar way as in the previous analysis, with the difference that they were placed in the same cluster (green horizontal cluster) with the CO_2_-NADES systems monitored at the start of the experiment (Gly/Glu0). The remaining CO_2_-NADES systems were placed in a separate cluster (red and purple horizontal cluster).

**Figure 4:**
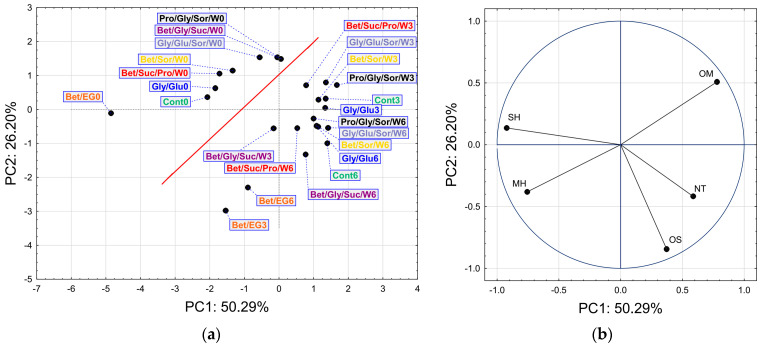
The score plot (**a**) and loadings plot (**b**) of the PCA of NADES based on headspace composition. (OM—Oxygenated Monoterpenes, MH—Monoterpene Hydrocarbons, NT—Non-Terpenes, OS—Oxygenated Sesquiterpenes, SH—Sesquiterpene Hydrocarbons).

The PCA modeling, based on the chemical composition in terms of the detected group of compounds, resulted in a model that took into account 76.5% of total variance based on two PCs described by Eigenvalues (Ei) higher than 1: PC1 (Ei = 2.51) explained 50.29%, and PC2 (Ei = 1.31) explained 26.20% of the total variance. In the score plot, presented in Figure 4a, it is evident that there was a clear separation (red line) between the samples monitored at the start of the experiment and the samples monitored after 3 and 6 months. The distribution of the samples along the PC1 axis was mostly influenced by SH, MH, and OM content, whereas the OS content had a significant influence on the PC2 axis (the score plot, Figure 4b). The content of NT had a moderate influence on the distribution of the compounds along both the PC1 and PC2 axes. The PCA results were in agreement with the results obtained by HCA presented in Figure 3. The main observations arising from the score graph indicated the distinction of the Bet/EG samples and significant differences between the control samples (significant distance of the sample Cont0 from the other control samples is evident). Additionally, the score plot indicated a noticeable change in the content of certain groups of compounds (SH, MH, and OM) from 0 to 3 months for certain samples (e.g., control samples, Bet/EG, Gly/Glu, Bet/Suc/Pro/W, Bet/Sor/W).

### 3.2. The Ranking of the Samples Based on the SRD Approach

**Figure 5:**
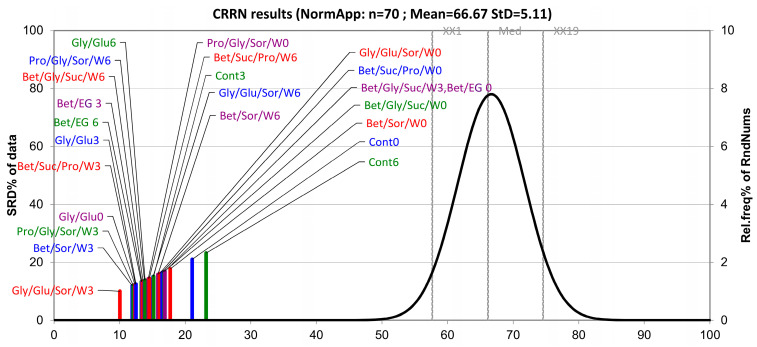
The ranking of the NADES by SRD and CRRN with row average as a reference ranking. The right *y*-axis presents the relative frequencies for the Gauss curve (XX1 = 5% limit, Med = Median, XX19 = 95% limit).

The ranking of the samples in terms of the order of observations (abundance of each detected compound) was carried out based on row average ranking as a golden standard (consensus ranking). The obtained results are presented in Figure 5. Based on the results, it can be concluded that there was no particular grouping of the samples regarding their SRD values. However, it can be noticed that the sample Gly/Glu/Sor/W3 was closest to the reference ranking, and the control samples Cont0 and Cont6 were placed furthest from the reference ranking, whereas the Cont3 sample was placed among the other CO_2_-NADES samples. This implied that the control samples Cont0 and Cont6 had a significantly different order of the compounds than the reference ranking and CO_2_-NADES systems. This could also imply that the variations in the change in composition in the control samples were more pronounced than in the case of the CO_2_-NADES samples. All the samples were significantly distant from the Gaussian curve, indicating that the results were not random in nature.

**Figure 6:**
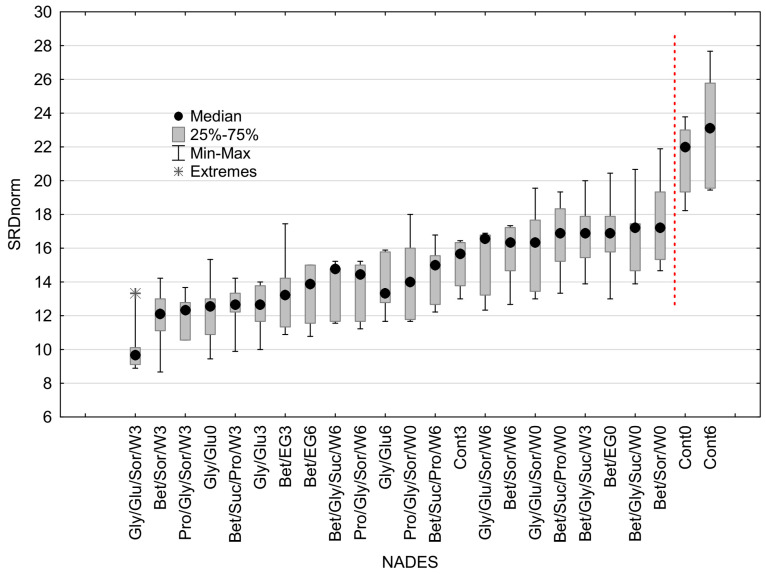
The results of 7-fold cross-validation of the SRD procedure.

The results of 7-fold cross-validation of the SRD procedure are presented in Figure 6 in the form of a Box–Whisker plot. The results are based on normalized SRD values (SRDnorm) obtained in each repeated ranking procedure.

The CO_2_-NADES and control samples were ordered in the same way as in the SRD graph (Figure 5). For each sample, there were seven SRD values obtained for each repeated SRD analysis. The median, 25%, and 75% of the range, minimum, and maximum values are presented as a Box–Whisker plot. The graph points to the consistency in the SRD analysis of the samples. An extreme SRDnorm value is observable for the sample Gly/Glu/Sor/W3; however, it can be considered insignificant. The vertical red dashed line shows the significant difference between the Cont0 and Cont6 samples in comparison to the other samples based on the Wilcoxon Matched Pairs Test at the 0.05 confidence level. This is a confirmation of the previously mentioned assumption that Cont0 and Cont6 possessed a significantly different order of the compounds content than the reference ranking and CO_2_-NADES samples.

### 3.3. Wilcoxon Matched Pairs Test of the NADES Samples

Pairwise comparison of samples observed at the beginning of the experiment and after 3 and 6 months was performed using the Wilcoxon Matched Pairs Test to test whether the mean values of the samples differed over time (Table 5). The significant composition change between 3 and 6 months was noticeable for the control, Bet/Suc/Pro/W, Bet/Gly/Suc/W, Pro/Gly/Sor/W, and Gly/Glu/Sor/W samples. The Wilcoxon test implied the stability of the Gly/Glu, Bet/Sor/W and Bet/EG samples for 6 months.

The improved stability of the components dispersed in individual NADES compared to the control after 6 months of storage at room temperature is evident. A possible explanation for the better stability of the CO_2_–NADES systems compared to the control is that NADES played the role of a physical protector of the components, which, consequently, ensured the protection of the components from oxidative degradation. Potentially, the high viscosity of Gly/Glu (5258.47 ± 322.86 mPa·s) and Bet/Gly/Suc/W (2246.77 ±45.27 mPa·s) could be one of the factors that contributed to the stabilization. In a previous study, it had been suggested that stabilization in high-viscous NADES can contribute to the preservation due to the reduced movement of the molecules [21]. However, in our study, no clear relationship between viscosity and stability was noticed because Bet/EG (48.63 ± 0.42 mPa·s) with a very low viscosity proved to be one of the NADES with stabilizing capacity. Furthermore, the stabilizing capacity of NADES may be due to the development of intermolecular interactions with individual components of *S. montana* [11,21]. Namely, *S. montana* components can play the role of hydrogen bond donors and acceptors; hence, they can potentially form interactions with NADES components and thus slow down the movement of molecules, preventing oxidation and other degradation reactions and maintaining the overall stability. Also, for the further implementation of the established process on a large scale, an important aspect is the price of the used NADES (Table 1). Amino-acid-based NADES are evidently more expensive compared to sugar-based ones. Among the systems for which stability was established over time, the cheapest was Bet/Sor/W, followed by Bet/EG and Gly/Glu.

Conventional procedures for obtaining and stabilizing aroma volatile components include Soxhlet or hydrodistillation, followed by spray drying. Soxhlet extraction involves the use of hazardous organic solvents, as well as the possibility of their remaining in the final product. On the other hand, hydrodistillation is performed at high temperatures that can lead to the degradation of thermally labile compounds, and, as a result, the smell of hydrodistilled essential oil is often not authentic to the source. With the application of supercritical CO_2_ at mild temperatures, the possibility of deterioration is reduced, there is no use or generation of toxic waste, and the obtained extract is clean and safe [42]. Furthermore, the spray drying technique is the most common method of preserving volatile components; however, it implies the use of carriers and additional processing of the sample, which, apart from additional costs, also suggests working at high temperatures and the possibility of losing thermo-sensitive components [5]. On the other hand, the preparation of NADES is simple, and dispersion in NADES avoids additional processing of samples at high temperature.

The resulting systems represent products with numerous potential applications. Due to the presence of components with important pharmacological properties, such as carvacrol, thymol, thymoquinone, and others, they can be used in obtaining pharmaceutical products. Also, the components of *S. montana* are highly relevant in the cosmetic industry as well as the perfume industry. Additionally, the properties of *S. montana* components, such as the improvement of sensory characteristics and preservative and antimicrobial properties, are also important for the food industry. Moreover, the obtained systems can play an important role in creating repellent products [43,44].

## 4. Conclusions

The approach investigated in this work represents the sustainable aroma stabilization process, which ensures the efficient attainment of aroma compounds and their preservation, consequently reducing costs, promoting a more rational use of natural resources, and reducing waste generation. Also, the goals of this work correspond to the objectives of green chemistry and the Sustainable Development Goals agenda, as they include environmental and economic sustainability, environmental protection, and human well-being.

By comparison, between the changes in the headspace profiles of the control and CO_2_-NADES systems, it is evident that the application of NADES advanced the stability of *S. montana* volatile compounds. Apart from the more pronounced changes with regard to oxidation and other transformational reactions, the formation of non-terpene components such as acetic acid, E,E-hepta-2,4-dienal, and γ-butyrolactone was observed in the control. Moreover, these components can significantly reduce the quality of the products. In CO_2_-NADES systems, there was no formation of these components, and the changes in the headspace profiles were less pronounced compared to the control. Bet/EG was observed as a system with the lowest oscillations over time. In addition, using the Wilcoxon test, it was established that the systems with the least changes were Bet/EG, Gly/Glu, and Bet/Sor/W.

It is of great importance to continue the study of aroma compounds from different types of materials and their interactions with different NADES because the stabilities of volatile components, essential oils, extracts, and other products containing volatile aroma compounds depend on the characteristics of those components and their tendencies to oxidation and degradation changes.

## Figures and Tables

**Table 1:** Composition, molar ratio, abbreviation, and viscosity of the prepared NADESs.

NADES Composition	Molar Ratio	Abbreviation	Price (EUR kg^−1^) ^a^	Viscosity (mPa·s) 30 °C
Betaine/Ethylene glycol	1:3	Bet/EG	95.34	48.63 ± 0.42
Betaine/Sorbitol/Water	1:1:3	Bet/Sor/W	81.07	747.40 ± 14.83
Betaine/Sucrose/Proline/Water	5:2:2:21	Bet/Suc/Pro/W	170.09	853.70 ± 39.37
Betaine/Glycerol/Sucrose/Water	2:3:1:5	Bet/Gly/Suc/W	109.47	2246.77 ± 45.27
Glycerol/Glucose	4:1	Gly/Glu	118.13	5258.47 ± 322.86
Glycerol/Glucose/Sorbitol/Water	1:1:1:3	Gly/Glu/Sorb/W	68.95	1460.03 ± 53.23
Glycerol/Proline/Sorbitol/Water	1:1:1:13	Pro/Gly/Sorb/W	190.55	23.70 ± 1.44

^a^—The price of systems was estimated according to the website of Merck (Germany) for individual components. The price of proline was estimated according to the website of Alfa Aesar (Germany; United States).

**Table 2:** The volatile headspace profile (%) of *Satureja montana* extracted by headspace solid-phase microextraction and analyzed by gas chromatography–mass spectrometry at the start (month 0).

Compound	RI	Sample							
		Control	Glyc/Glu	Bet/Suc/Pro/W	Bet/Gly/Suc/W	Bet/Sor/W	Pro/Gly/Sor/W	Gly/Glu/Sor/W	Bet/EG
Monoterpene hydrocarbons									
*α*-Pinene	940	0.09 ± 0.01		0.02 ± 0.00	0.06 ± 0.02	0.03 ± 0.01	0.01 ± 0.00	0.01 ± 0.01	0.02 ± 0.01
Camphene	955	0.20 ± 0.00		0.05 ± 0.03	0.03 ± 0.01	0.06 ± 0.02	0.01 ± 0.00	0.04 ± 0.02	0.11 ± 0.07
*β*-Myrcene	993	0.38 ± 0.07	0.08 ± 0.01	0.32 ± 0.19	0.24 ± 0.19	0.25 ± 0.12	0.13 ± 0.07	0.22 ± 0.05	0.16 ± 0.08
*α*-Phellandrene	1011	0.26 ± 0.11	0.06 ± 0.02	0.26 ± 0.10	0.24 ± 0.12	0.20 ± 0.08	0.15 ± 0.05	0.19 ± 0.04	0.08 ± 0.01
*α*-Terpinene	1023	0.73 ± 0.09	0.16 ± 0.06	0.41 ± 0.29	0.36 ± 0.21	0.36 ± 0.04	0.21 ± 0.10	0.33 ± 0.13	0.31 ± 0.12
*p*-Cymene	1030	5.61 ± 0.42	2.47 ± 0.88	2.79 ± 0.28	2.56 ± 1.54	2.51 ± 0.88	1.11 ± 0.32	1.81 ± 0.81	7.00 ± 0.55
Limonene	1035	0.55 ± 0.05	0.16 ± 0.04	0.42 ± 0.09	0.35 ± 0.14	0.33 ± 0.09	0.21 ± 0.19	0.29 ± 0.14	0.29 ± 0.11
(Z)-*β*-Ocimene	1043	0.18 ± 0.06	0.05 ± 0.01	0.13 ± 0.04	0.11 ± 0.01	0.10 ± 0.01	0.06 ± 0.01	0.10 ± 0.04	0.08 ± 0.02
(E)-*β*-Ocimene	1054	0.22 ± 0.02	0.01 ± 0.00	0.20 ± 0.10	0.13 ± 0.10	0.16 ± 0.14	0.06 ± 0.02	0.14 ± 0.07	0.02 ± 0.01
*γ*-Terpinene	1065	0.88 ± 0.36	0.28 ± 0.08	0.47 ± 0.19	0.34 ± 0.08	0.38 ± 0.09	0.21 ± 0.09	0.31 ± 0.18	0.57 ± 0.05
Oxygenated monoterpenes									
Verbenene	968	0.08 ± 0.01		0.09 ± 0.01	0.08 ± 0.02	0.06 ± 0.01	0.02 ± 0.01	0.01 ± 0.00	0.02 ± 0.01
1,8-Cineole	1037	0.33 ± 0.15	0.15 ± 0.04	0.17 ± 0.08	0.14 ± 0.02	0.15 ± 0.05	0.10 ± 0.00	0.13 ± 0.02	0.29 ± 0.14
trans-Linalool oxide	1078	0.12 ± 0.08	0.05 ± 0.02	0.05 ± 0.01	0.06 ± 0.01	0.05 ± 0.01	0.05 ± 0.01	0.05 ± 0.01	0.05 ± 0.00
Linalool	1103	1.67 ± 1.03	1.21 ± 0.25	2.11 ± 0.32	1.81 ± 0.33	1.53 ± 0.22	1.51 ± 0.14	1.29 ± 0.11	1.50 ± 0.25
*α*-Thujone	1104	0.08 ± 0.02	0.06 ± 0.01	0.08 ± 0.02	0.06 ± 0.01	0.06 ± 0.01	0.05 ± 0.02	0.05 ± 0.00	0.10 ± 0.04
*β*-Thujone	1114	0.03 ± 0.00		0.03 ± 0.02					
Camphor	1150	0.86 ± 0.12	0.57 ± 0.28	0.90 ± 0.19	0.77 ± 0.15	0.72 ± 0.14	0.64 ± 0.24	0.62 ± 0.28	0.72 ± 0.23
Menthone	1160	0.44 ± 0.11	0.35 ± 0.15	0.46 ± 0.02	0.39 ± 0.18	0.33 ± 0.21	0.30 ± 0.17	0.28 ± 0.08	0.57 ± 0.12
Borneol	1170	4.13 ± 1.35	2.19 ± 0.88	4.90 ± 0.41	4.60 ± 1.89	3.74 ± 0.71	3.86 ± 0.17	3.93 ± 0.41	2.15 ± 0.58
Menthol	1171	0.87 ± 0.18	0.66 ± 0.14	1.27 ± 0.28	1.16 ± 0.88	0.87 ± 0.19	0.99 ± 0.22	0.86 ± 0.14	0.65 ± 0.17
Terpinene-4-ol	1182	4.22 ± 1.23	2.75 ± 0.25	5.14 ± 0.54	4.87 ± 1.26	3.81 ± 0.88	3.83 ± 1.21	3.70 ± 0.98	3.33 ± 0.87
*p*-Cymen-8-ol	1190	0.27 ± 0.12	0.23 ± 0.8	0.26 ± 0.11	0.32 ± 0.12	0.25 ± 0.04	0.35 ± 0.17	0.24 ± 0.14	0.16 ± 0.02
*α*-Terpineol	1194	0.46 ± 0.27	0.32 ± 0.10	0.61 ± 0.28	0.63 ± 0.14	0.46 ± 0.15	0.47 ± 0.11	0.43 ± 0.05	0.29 ± 0.09
*cis*-Dihydrocarvone	1199	0.16 ± 0.04	0.09 ± 0.02	0.13 ± 0.04	0.17 ± 0.04	0.15 ± 0.03	0.17 ± 0.04	0.10 ± 0.07	0.08 ± 0.02
*trans*-Dihydrocarvone	1207	0.07 ± 0.03		0.04 ± 0.01	0.06 ± 0.01				
Verbenone	1224	0.08 ± 0.02	0.02 ± 0.01	0.09 ± 0.01	0.09 ± 0.03	0.07 ± 0.04	0.07 ± 0.02	0.05 ± 0.01	0.07 ± 0.03
trans-Carveol	1230	0.06 ± 0.01	0.01 ± 0.01	0.07 ± 0.00	0.06 ± 0.01	0.05 ± 0.01	0.06 ± 0.01	0.05 ± 0.01	0.02 ± 0.01
Thymol methyl ether	1241	0.59 ± 0.21	0.46 ± 0.13	2.00 ± 0.88	0.47 ± 0.31	0.44 ± 0.11	0.25 ± 0.06	0.39 ± 0.09	1.16 ± 0.51
Cuminal	1246	0.29 ± 0.20	0.14 ± 0.04	0.36 ± 0.13	0.36 ± 0.18	0.29 ± 0.05	0.29 ± 0.03	0.24 ± 0.10	0.31 ± 0.28
Carvacrol methyl ether	1250	7.43 ± 2.69	6.24 ± 1.25	7.48 ± 2.02	6.51 ± 2.46	6.03 ± 1.04	4.05 ± 0.78	5.16 ± 1.05	13.15 ± 2.54
Thymoquinone	1258	12.84 ± 3.49	10.56 ± 1.67	8.82 ± 1.98	15.33 ± 2.06	12.14 ± 1.06	9.00 ± 2.24	11.78 ± 1.68	16.38 ± 0.92
Bornyl acetate	1297		0.67 ± 0.13	0.51 ± 0.80					0.96 ± 0.35
Thymol	1297	16.86 ± 2.97	16.25 ± 2.57	17.93 ± 1.68	20.84 ± 2.68	19.05 ± 0.47	23.48 ± 1.77	21.89 ± 1.92	5.65 ± 0.36
Carvacrol	1307	20.79 ± 4.59	19.96 ± 3.04	21.81 ± 2.16	25.56 ± 4.22	23.61 ± 1.28	29.37 ± 3.02	28.35 ± 2.07	6.38 ± 0.97
Dihydroactinidiolide	1532			0.11 ± 0.02	0.11 ± 0.04				
Sesquiterpene hydrocarbons									
*α*-Copaene	1378	0.32 ± 0.18	0.26 ± 0.04	0.30 ± 0.18	0.13 ± 0.03	0.25 ± 0.08	0.12 ± 0.04	0.21 ± 0.05	0.49 ± 0.25
*β*-Bourbonene	1386	0.68 ± 0.34	0.69 ± 0.12	0.65 ± 0.24	0.25 ± 0.05	0.52 ± 0.24	0.25 ± 0.13	0.42 ± 0.18	1.40 ± 0.88
*trans*-Caryophyllene	1419	5.35 ± 1.98	5.57 ± 1.38	6.12 ± 0.58	2.81 ± 1.52	5.09 ± 1.44	2.79 ± 0.77	4.42 ± 1.21	10.7 ± 0.69
*α*-Humulene	1456	0.77 ± 0.24	0.65 ± 0.14	1.10 ± 0.54	0.70 ± 0.12	1.06 ± 0.41	0.43 ± 0.18	0.86 ± 0.30	0.88 ± 0.22
*γ*-Muurolene	1477	0.58 ± 0.39	0.60 ± 0.23	0.79 ± 0.41	0.05 ± 0.01	0.04 ± 0.01	0.36 ± 0.22	0.08 ± 0.01	1.31 ± 0.68
Germacrene D	1482	0.55 ± 0.12	0.91 ± 0.14	0.89 ± 0.49	0.35 ± 0.20	0.69 ± 0.32	0.46 ± 0.18	0.51 ± 0.14	2.04 ± 0.87
ar-Curcumene	1483					0.05 ± 0.01			
*β*-Selinene	1486	0.21 ± 0.05	0.24 ± 0.08	0.27 ± 0.04	0.19 ± 0.10	0.28 ± 0.02	0.18 ± 0.11	0.28 ± 0.14	0.34 ± 0.14
Ledene	1493	0.98 ± 0.41	1.18 ± 0.41	1.34 ± 0.09	0.74 ± 0.04	1.14 ± 0.37	0.87 ± 0.51	0.97 ± 0.26	1.99 ± 0.63
*α*-Muurolene	1499	0.58 ± 0.29	0.52 ± 0.18	0.97 ± 0.17	0.83 ± 0.24	0.96 ± 0.18	0.73 ± 0.13	0.84 ± 0.15	0.73 ± 0.14
*β*-Bisabolene	1500	1.85 ± 0.46	2.08 ± 0.56	2.36 ± 0.95	1.15 ± 0.88	2.07 ± 0.56	0.94 ± 0.11	1.73 ± 0.34	4.06 ± 1.69
*γ*-Cadinene	1505	0.65 ± 0.18	0.74 ± 0.15	0.95 ± 0.18	0.50 ± 0.16	0.89 ± 0.12	0.52 ± 0.30	0.67 ± 0.15	1.37 ± 0.37
Δ-Cadinene	1525	1.07 ± 0.56	1.39 ± 0.23	1.44 ± 0.55	0.74 ± 0.22	1.28 ± 0.04	0.70 ± 0.19	1.07 ± 0.24	2.46 ± 0.84
*α*-Calacorene	1546	0.12 ± 0.08	0.16 ± 0.05	0.20 ± 0.02	0.11 ± 0.05	0.10 ± 0.02	0.12 ± 0.04	0.16 ± 0.04	0.26 ± 0.12
Oxygenated sesquiterpenes									
Spathulenol	1581	0.17 ± 0.03	1.46 ± 0.47	0.87 ± 0.12	0.84 ± 0.27	0.83 ± 0.62	1.26 ± 0.36	0.64 ± 0.08	0.57 ± 0.14
Others									
Acetic acid	<900	0.01 ± 0.00							
Oct-1-en-3-ol	986	0.80 ± 0.26	0.40 ± 0.12	0.66 ± 0.18	0.61 ± 0.18	0.51 ± 0.17	0.52 ± 0.14	0.54 ± 0.14	0.42 ± 0.04
6-Methyl-5-hepten-2-one	988	0.03 ± 0.01							
Octan-3-one	989	0.07 ± 0.01							
2-Phenylethanol	1112	0.15 ± 0.07						0.06 ± 0.03	

**Table 3:** The volatile headspace profile (%) of *Satureja montana* extracted by headspace solid-phase microextraction and analyzed by gas chromatography–mass spectrometry after 3 months of storage.

Compound	RI	Sample							
		Control	Glyc/Glu	Bet/Suc/Pro/W	Bet/Gly/Suc/W	Bet/Sor/W	Pro/Gly/Sor/W	Gly/Glu/Sor/W	Bet/EG
Monoterpene hydrocarbons									
*α*-Pinene	940	0.04 ± 0.01	0.05 ± 0.02	0.07 ± 0.02	0.05 ± 0.04	0.07 ± 0.05	0.02 ± 0.01	0.03 ± 0.02	0.15 ± 0.02
Camphene	955	0.08 ± 0.03	0.09 ± 0.02	0.13 ± 0.06	0.04 ± 0.01	0.12 ± 0.05	0.05 ± 0.02	0.06 ± 0.02	0.28 ± 0.10
*β*-Myrcene	993								0.07 ± 0.01
*α*-Phellandrene	1011								0.05 ± 0.00
*α*-Terpinene	1023	0.10 ± 0.02		0.03 ± 0.01	0.05 ± 0.01	0.07 ± 0.02	0.01 ± 0.00	0.07 ± 0.01	0.14 ± 0.04
*p*-Cymene	1030	2.49 ± 0.65	2.79 ± 0.41	3.05 ± 0.44	7.32 ± 3.04	2.68 ± 0.58	0.84 ± 0.17	1.80 ± 0.41	8.65 ± 2.58
Limonene	1035	0.22 ± 0.04	0.15 ± 0.03	0.14 ± 0.02	0.12 ± 0.03	0.19 ± 0.03			
*γ*-Terpinene	1065	0.09 ± 0.03		0.04 ± 0.02	0.05 ± 0.01	0.06 ± 0.02	0.09 ± 0.03	0.07 ± 0.04	0.04 ± 0.01
Oxygenated monoterpenes									
1,8-Cineole	1037	0.40 ± 0.09	0.54 ± 0.12	0.54 ± 0.21	0.11 ± 0.02	0.43 ± 0.22	0.35 ± 0.10	0.39 ± 0.12	0.07 ± 0.03
trans-Linalool oxide	1078	0.20 ± 0.08				0.40 ± 0.15	0.17 ± 0.08	0.21 ± 0.05	
Linalool	1103	2.74 ± 0.45	3.21 ± 0.22	4.39 ± 0.78	3.84 ± 1.75	3.86 ± 1.31	3.34 ± 0.20	2.79 ± 0.41	2.76 ± 0.87
Camphor	1150	1.20 ± 0.31	1.01 ± 0.06	1.44 ± 0.39	1.26 ± 0.12	1.30 ± 0.88	1.23 ± 0.23	0.98 ± 0.61	1.14 ± 0.56
Menthone	1160	0.34 ± 0.12	0.11 ± 0.04	0.05 ± 0.01	0.04 ± 0.01	0.41 ± 0.18	0.34 ± 0.04	0.29 ± 0.12	0.60 ± 0.23
Borneol	1170	4.63 ± 1.23	3.59 ± 0.83	6.07 ± 1.05	4.93 ± 1.54	5.49 ± 0.71	5.90 ± 0.71	4.94 ± 0.15	2.80 ± 0.48
Menthol	1171		0.22 ± 0.15	0.23 ± 0.06	1.32 ± 0.44	0.48 ± 0.23	0.78 ± 0.12	0.68 ± 0.14	0.78 ± 0.15
Terpinene-4-ol	1182	6.79 ± 2.60	6.28 ± 1.36	8.29 ± 1.28	7.29 ± 2.04	7.32 ± 0.47	8.41 ± 2.47	6.44 ± 2.51	5.95 ± 0.56
*p*-Cymen-8-ol	1190		0.26 ± 0.14	0.12 ± 0.05	0.28 ± 0.12	0.42 ± 0.04	0.19 ± 0.04	0.32 ± 0.05	0.29 ± 0.14
*α*-Terpineol	1194	0.84 ± 0.06	0.52 ± 0.15	0.54 ± 0.04	0.22 ± 0.04	0.65 ± 0.23	0.22 ± 0.10	0.12 ± 0.10	0.33 ± 0.10
*cis*-Dihydrocarvone	1199		0.01 ± 0.01		0.21 ± 0.09	0.09 ± 0.01		0.09 ± 0.06	0.10 ± 0.02
Verbenone	1224		0.01	0.03 ± 0.01	0.12 ± 0.01	0.03 ± 0.02		0.01 ± 0.00	0.02 ± 0.01
*trans*-Carveol	1230	0.01 ± 0.01		0.05 ± 0.02	0.10 ± 0.01	0.01 ± 0.01	0.07 ± 0.01	0.01 ± 0.00	0.01 ± 0.00
Thymol methyl ether	1241	0.24 ± 0.14	0.09 ± 0.03	0.10 ± 0.02	0.11 ± 0.03	0.19 ± 0.11	0.08 ± 0.02	0.14 ± 0.04	0.03 ± 0.01
Cuminal	1246			0.14 ± 0.08	0.15 ± 0.05	0.18 ± 0.12		0.12 ± 0.03	0.29 ± 0.14
Carvacrol methyl ether	1250	3.75 ± 1.54	4.95 ± 0.87	8.96 ± 1.41	7.03 ± 2.87	3.98 ± 0.96	2.98 ± 0.41	3.18 ± 0.58	9.44 ± 1.58
Thymoquinone	1258	12.51 ± 2.08	15.39 ± 2.05	8.96 ± 1.69	12.58 ± 3.16	13.17 ± 1.58	7.26 ± 1.41	12.46 ± 2.27	16.64 ± 2.66
Bornyl acetate	1297	0.39 ± 0.07		0.13 ± 0.04		0.37 ± 0.10	0.35 ± 0.10	0.35 ± 0.12	0.77 ± 0.14
Thymol	1297	20.38 ± 3.15	20.67 ± 0.47	18.36 ± 2.12	16.67 ± 1.89	19.12 ± 1.06	22.94 ± 1.89	22.17 ± 1.36	8.71 ± 0.57
Carvacrol	1307	28.95 ± 2.08	27.34 ± 1.51	25.31 ± 0.25	22.19 ± 0.87	25.50 ± 2.71	31.44 ± 3.21	29.72 ± 2.14	11.61 ± 0.66
Dihydroactinidiolide	1532	0.04 ± 0.01		0.08 ± 0.04	0.10 ± 0.06	0.09 ± 0.02		0.39 ± 0.16	
*α*-Copaene	1378	0.13 ± 0.06				0.16 ± 0.09	0.03 ± 0.00	0.10 ± 0.08	0.35 ± 0.12
*β*-Bourbonene	1386	0.37 ± 0.09	0.23 ± 0.05	0.10 ± 0.04	0.11 ± 0.01	0.41 ± 0.17	0.29 ± 0.09	0.28 ± 0.11	1.00 ± 0.33
trans-Caryophyllene	1419	2.58 ± 0.21	0.93 ± 0.41	4.23 ± 0.09	2.83 ± 0.81	1.93 ± 0.47	2.51 ± 0.88	2.00 ± 0.36	2.93 ± 0.74
*α*-Humulene	1456	0.18 ± 0.04		0.15 ± 0.09		0.13 ± 0.04	0.10 ± 0.01	0.13 ± 0.07	0.39 ± 0.14
*γ*-Muurolene	1477	0.27 ± 0.18	0.16 ± 0.04	0.07 ± 0.06		0.11 ± 0.03	0.25 ± 0.15	0.19 ± 0.08	0.76 ± 0.26
Germacrene D	1482	0.12 ± 0.01				0.12 ± 0.04	0.23 ± 0.03	0.06 ± 0.02	
*β*-Selinene	1486					0.17 ± 0.05			0.30 ± 0.10
Ledene	1493	0.12 ± 0.03						0.13 ± 0.06	
*α*-Muurolene	1499	0.04 ± 0.01		0.13 ± 0.01		0.07 ± 0.02		0.05 ± 0.01	0.01 ± 0.00
*β*-Bisabolene	1500	1.41 ± 0.31	1.19 ± 0.62	1.48 ± 0.27	2.08 ± 0.19	1.13 ± 0.14	0.79 ± 0.14	1.14 ± 0.54	1.91 ± 0.87
*γ*-Cadinene	1505	0.29 ± 0.06	0.22 ± 0.02	0.28 ± 0.13		0.17 ± 0.02	0.06 ± 0.02	0.21 ± 0.12	0.65 ± 0.14
Δ-Cadinene	1525	0.46 ± 0.08	0.45 ± 0.58	0.15 ± 0.07	0.62 ± 0.14	0.35 ± 0.17	0.36 ± 0.12	0.43 ± 0.18	0.89 ± 0.33
*α*-Calacorene	1546		0.11 ± 0.10				0.08 ± 0.01		0.18 ± 0.12
Oxygenated sesquiterpenes									
Spathulenol	1581		0.92 ± 0.14	0.16 ± 0.02	1.00 ± 0.15	0.23 ± 0.03	1.01 ± 0.18	0.92 ± 0.17	1.23 ± 0.48
Caryophyllene oxide	1581	3.54 ± 0.95	6.56 ± 1.86	3.45 ± 0.74	4.91 ± 2.66	5.37 ± 0.66	4.84 ± 1.24	4.09 ± 0.74	12.04 ± 1.97
Others									
Acetic acid	<900	1.29 ± 0.28							
Oct-1-en-3-ol	986	1.02 ± 0.40	0.86 ± 0.15	1.30 ± 0.31	1.10 ± 0.14	1.29 ± 0.41	1.13 ± 0.18	1.03 ± 0.38	0.65 ± 0.39
Octan-3-one	989	0.07 ± 0.02	0.06 ± 0.01	0.04 ± 0.02	0.02 ± 0.01	0.01 ± 0.00	0.01 ± 0.01		
2-Phenylethanol	1112	0.07 ± 0.01							

**Table 4:** The volatile headspace profile (%) of *Satureja montana* extracted by headspace solid-phase microextraction and analyzed by gas chromatography–mass spectrometry after 6 months of storage.

Compound	RI	Sample							
		Control	Glyc/Glu	Bet/Suc/Pro/W	Bet/Gly/Suc/W	Bet/Sor/W	Pro/Gly/Sor/W	Gly/Glu/Sor/W	Bet/EG
Monoterpene hydrocarbons									
α-Pinene	940	0.05 ± 0.02			0.05 ± 0.01			0.03 ± 0.02	0.16 ± 0.08
Camphene	955	0.25 ± 0.04		0.09 ± 0.03	0.09 ± 0.03	0.08 ± 0.04	0.04 ± 0.03	0.09 ± 0.02	0.28 ± 0.14
*α*-Phellandrene	1011	0.06 ± 0.01		0.09 ± 0.02		0.08 ± 0.01	0.10 ± 0.04	0.08 ± 0.05	0.06 ± 0.01
*α*-Terpinene	1023	0.16 ± 0.08	0.11 ± 0.04	0.13 ± 0.08	0.11 ± 0.04	0.09 ± 0.03	0.10 ± 0.02	0.14 ± 0.04	0.18 ± 0.05
*p*-Cymene	1030	2.58 ± 0.18	1.18 ± 0.35	1.84 ± 0.26	3.06 ± 1.32	1.61 ± 0.19	1.46 ± 0.74	2.48 ± 1.14	6.38 ± 0.63
Limonene	1035	0.13 ± 0.03	0.12 ± 0.04	0.12 ± 0.01	0.16 ± 0.04	0.10 ± 0.07			
*γ*-Terpinene	1065	0.03 ± 0.01	0.05 ± 0.01	0.11 ± 0.04	0.05 ± 0.01	0.05 ± 0.01	0.08 ± 0.04	0.04 ± 0.01	0.11 ± 0.07
Oxygenated monoterpenes									
1,8-Cineole	1037	0.59 ± 0.25	0.11 ± 0.03	0.31 ± 0.26	0.32 ± 0.14	0.28 ± 0.04	0.37 ± 0.14	0.39 ± 0.25	0.70 ± 0.28
*cis*-Linalool oxide	1087								
*trans*-Linalool oxide	1078	0.29 ± 0.10	0.14 ± 0.05	0.20 ± 0.15	0.18 ± 0.05	0.22 ± 0.05	0.22 ± 0.07	0.21 ± 0.17	0.10 ± 0.04
Linalool	1103	3.14 ± 1.03	3.40 ± 1.04	4.57 ± 1.39	3.49 ± 1.99	3.75 ± 0.94	3.78 ± 0.51	3.19 ± 0.96	3.13 ± 0.68
*α*-Thujone	1104	0.13 ± 0.04	0.08 ± 0.02	0.07 ± 0.02	0.10 ± 0.02	0.10 ± 0.02	0.10 ± 0.01	0.12 ± 0.04	0.17 ± 0.12
*cis*-*p*-Mentha-2-en-1-ol	1126	0.25 ± 0.06	0.09 ± 0.04	0.28 ± 0.12	0.26 ± 0.04	0.28 ± 0.10	0.27 ± 0.08	0.14 ± 0.08	0.13 ± 0.09
*β*-Thujone	1114			0.06 ± 0.01					
Camphor	1150	1.25 ± 0.15	0.91 ± 0.42	1.35 ± 0.24	1.12 ± 0.45	1.15 ± 0.09	1.24 ± 0.61	1.14 ± 0.31	1.32 ± 0.54
Menthone	1160	0.48 ± 0.13	0.31 ± 0.15	0.49 ± 0.12	0.47 ± 0.18	0.41 ± 0.19	0.46 ± 0.14	0.42 ± 0.12	0.70 ± 0.24
Borneol	1170	4.85 ± 1.39	3.73 ± 0.65	5.61 ± 0.54	4.56 ± 2.04	4.94 ± 1.04	5.43 ± 0.98	4.84 ± 1.58	3.64 ± 1.87
Menthol	1171	1.47 ± 0.58	1.58 ± 0.41	2.11 ± 0.21	1.66 ± 0.85	1.75 ± 0.66	1.90 ± 0.41	1.59 ± 0.69	1.32 ± 0.65
Terpinene-4-ol	1182	5.73 ± 1.52	5.49 ± 1.58	6.72 ± 1.08	5.41 ± 1.24	5.67 ± 0.71	6.55 ± 0.24	5.35 ± 0.45	5.98 ± 1.00
*p*-Cymen-8-ol	1190	1.16 ± 0.26	0.37 ± 0.12	1.10 ± 0.05	0.86 ± 0.04	0.99 ± 0.21	1.04 ± 0.12	0.93 ± 0.31	0.43 ± 0.05
Terpenediol I	1192	0.05 ± 0.01							
*α*-Terpineol	1194	0.97 ± 0.22	0.81 ± 0.28	1.41 ± 0.78	1.00 ± 0.21	1.20 ± 0.14	1.14 ± 0.27	0.96 ± 0.41	0.67 ± 0.24
*cis*-Dihydrocarvone	1199	0.20 ± 0.06	0.07 ± 0.02	0.27 ± 0.02	0.47 ± 0.12	0.23 ± 0.02	0.21 ± 0.08	0.18 ± 0.08	0.20 ± 0.05
*trans*-Dihydrocarvone	1207	0.04 ± 0.01							
Verbenone	1224	0.31 ± 0.15	0.05 ± 0.01	0.15 ± 0.04	0.21 ± 0.10	0.15 ± 0.04	0.04 ± 0.01	0.08 ± 0.02	0.07 ± 0.04
*trans*-Carveol	1230			0.21 ± 0.08	0.15 ± 0.05	0.20 ± 0.02	0.22 ± 0.07	0.22 ± 0.09	0.01 ± 0.00
Thymol methyl ether	1241	0.36 ± 0.14	0.30 ± 0.14	0.44 ± 0.17	0.56 ± 0.19	0.34 ± 0.11	0.33 ± 0.05	0.31 ± 0.17	0.77 ± 0.18
Cuminal	1246	0.31 ± 0.06	0.11 ± 0.08	0.26 ± 0.10	0.32 ± 0.11	0.31 ± 0.12	0.26 ± 0.05	0.25 ± 0.05	0.21 ± 0.10
Carvacrol methyl ether	1250	3.58 ± 1.58	3.99 ± 0.05	4.75 ± 0.88	5.57 ± 1.05	4.03 ± 0.31	3.45 ± 1.25	3.52 ± 0.28	7.91 ± 1.84
Thymoquinone	1258	13.50 ± 2.56	14.97 ± 1.04	6.62 ± 2.00	13.07 ± 3.58	14.52 ± 1.88	5.88 ± 0.48	12.85 ± 1.28	15.19 ± 0.84
Geraniol	1262	0.40 ± 0.16	0.74 ± 0.18	0.75 ± 0.18	0.82 ± 0.22	0.97 ± 0.31	1.04 ± 0.22	0.83 ± 0.14	0.70 ± 0.25
Piperitone	1263	0.13 ± 0.04	0.18 ± 0.04	0.06 ± 0.01	0.05 ± 0.01	0.13 ± 0.04	0.10 ± 0.02	0.09 ± 0.01	0.16 ± 0.07
Bornyl acetate	1297	0.52 ± 0.32	0.66 ± 0.32	0.71 ± 0.22	0.69 ± 0.14	0.67 ± 0.18	0.54 ± 0.14	0.55 ± 0.00	1.01 ± 0.15
Thymol	1297	15.41 ± 1.56	16.36 ± 2.58	15.84 ± 2.84	14.02 ± 3.14	15.13 ± 2.08	18.40 ± 1.44	17.62 ± 1.05	8.69 ± 1.65
Carvacrol	1307	18.68 ± 3.15	20.66 ± 1.88	19.45 ± 0.41	16.70 ± 2.50	18.66 ± 0.39	23.05 ± 1.84	21.48 ± 0.41	10.53 ± 2.84
Limonen-1,2-diol	1342	0.17 ± 0.05			0.17 ± 0.04	0.11 ± 0.08		0.07 ± 0.01	
Dihydroactinidiolide	1532	0.20 ± 0.10	0.13 ± 0.00	0.27 ± 0.11	0.24 ± 0.15	0.31 ± 0.07	0.29 ± 0.15	0.24 ± 0.04	0.11 ± 0.05
Sesquiterpene hydrocarbons									
*α*-Copaene	1378	0.17 ± 0.06	0.32 ± 0.12	0.31 ± 0.04	0.34 ± 0.12	0.20 ± 0.04	0.16 ± 0.05	0.14 ± 0.04	0.33 ± 0.14
*β*-Bourbonene	1386	0.48 ± 0.24	0.79 ± 0.24	0.92 ± 0.10	1.07 ± 0.17	0.66 ± 0.28	0.56 ± 0.14	0.45 ± 0.12	1.14 ± 0.35
*trans*-Caryophyllene	1419	2.23 ± 1.26	0.73 ± 0.42	4.55 ± 1.85	2.03 ± 1.54	1.39 ± 0.77	2.89 ± 0.88	1.80 ± 0.58	2.87 ± 0.12
*α*-Humulene	1456		0.11 ± 0.06	0.44 ± 0.14	0.26 ± 0.14	0.22 ± 0.11	0.34 ± 0.14	0.21 ± 0.14	0.45 ± 0.08
*γ*-Muurolene	1477		0.58 ± 0.21	0.11 ± 0.02	0.11 ± 0.01	0.12 ± 0.02	0.32 ± 0.05	0.28 ± 0.16	0.18 ± 0.02
Germacrene D	1482	0.13 ± 0.02	0.05 ± 0.01	0.37 ± 0.11	0.11 ± 0.03	0.16 ± 0.05	0.24 ± 0.07	0.08 ± 0.01	0.21 ± 0.05
ar-Curcumene	1483		0.05 ± 0.02	0.07 ± 0.02					
*β*-Selinene	1486	0.22 ± 0.07	0.10 ± 0.01	0.25 ± 0.12	0.29 ± 0.07	0.40 ± 0.09	0.69 ± 0.11		0.24 ± 0.09
Ledene	1493		0.10 ± 0.05						
*α*-Muurolene	1499	0.88 ± 0.35	0.11 ± 0.04	0.11 ± 0.07	0.17 ± 0.11	0.97 ± 0.27	0.11 ± 0.08	0.28 ± 0.08	0.57 ± 0.14
*β*-Bisabolene	1500	0.87 ± 0.28	1.62 ± 0.41	1.86 ± 0.05	2.14 ± 0.41	1.07 ± 0.64	0.82 ± 0.22	0.61 ± 0.14	1.62 ± 0.36
*γ*-Cadinene	1505	0.41 ± 0.11	0.77 ± 0.31	0.75 ± 0.06	0.78 ± 0.13	0.57 ± 0.29	0.63 ± 0.14	0.45 ± 0.25	
Δ-Cadinene	1525	0.69 ± 0.24	1.13 ± 0.78	1.08 ± 0.21	1.02 ± 0.22	0.69 ± 0.24	0.63 ± 0.42	0.56 ± 0.25	1.12 ± 0.44
*α*-Calacorene	1546	0.12 ± 0.04	0.22 ± 0.10	0.15 ± 0.04	0.11 ± 0.02	0.05 ± 0.01	0.14 ± 0.04	0.12 ± 0.01	0.21 ± 0.05
Oxygenated sesquiterpenes									
Spathulenol	1581	1.41 ± 0.66	2.06 ± 0.65	2.05 ± 0.11	1.97 ± 0.23	2.14 ± 0.41	2.31 ± 0.75	1.69 ± 0.14	2.32 ± 0.41
Caryophyllene oxide	1581	2.78 ± 1.15	5.07 ± 1.59	3.96 ± 0.54	5.38 ± 1.52	4.69 ± 0.67	3.58 ± 0.22	3.40 ± 0.64	7.76 ± 0.87
Veridiflorol	1592	0.27 ± 0.06	0.40 ± 0.13	0.34 ± 0.12	0.36 ± 0.14	0.40 ± 0.14	0.38 ± 0.04	0.29 ± 0.04	0.48 ± 0.01
Others									
Acetic acid	<900	1.45 ± 0.60							
*γ*-Butyrolactone	921	0.07 ± 0.02							
Oct-1-en-3-ol	986	1.45 ± 0.12	1.54 ± 0.62	1.77 ± 0.41	2.39 ± 0.76	1.43 ± 0.76	1.56 ± 0.81	2.42 ± 1.03	1.01 ± 0.02
(E,E)-Hepta-2,4-dienal	1016	0.13 ± 0.05							
5-Methyl-5-vinyldihydrofuran-2(3H)-one	1047	0.10 ± 0.03		0.04 ± 0.00					
4-Methyl-benzaldehyde	1086	0.18 ± 0.07		0.19 ± 0.09	0.15 ± 0.02	0.13 ± 0.09	0.07 ± 0.02	0.08 ± 0.05	
Octan-3-one	989	0.10 ± 0.01				0.15 ± 0.09	0.09 ± 0.04		0.11 ± 0.01
2-Phenylethanol	1112	0.26 ± 0.06	0.08 ± 0.03	0.16 ± 0.09	0.11 ± 0.06	0.12 ± 0.04	0.14 ± 0.02	0.15 ± 0.07	0.14 ± 0.06
Vanillin	1398	0.10 ± 0.01							
Eugenol	1359	0.04 ± 0.02						0.03 ± 0.00	
1,3,5-Trimethoxy-benzene	1410	0.13 ± 0.03	0.14 ± 0.05	0.18 ± 0.04	0.18 ± 0.07	0.14 ± 0.04	0.12 ± 0.05	0.19 ± 0.11	0.07 ± 0.03

**Table 5:** Wilcoxon matched pairs test results (*p* = 0.01)—the colored cells between the columns symbolize the statistically significant (red color) or non-significant (green color) difference between the samples.

Start of the Monitoring		Monitoring after 3 Months		Monitoring after 6 Months
Cont0		Cont3		Cont6
	*N _valid_* = 54 *T* = 476.5 *Z* = 2.290 *p* = 0.022		*N _valid_* = 62 *T* = 500.5 *Z* = 3.337 *p* = 0.001	
Gly/Glu0		Gly/Glu3		Gly/Glu6
	*N _valid_* = 48 *T* = 472.5 *Z* = 1.185 *p* = 0.236		*N _valid_* = 51 *T* = 423.5 *Z* = 2.245 *p* = 0.025	
Bet/Suc/Pro/W0		Bet/Suc/Pro/W3		Bet/Suc/Pro/W6
	*N _valid_* = 52 *T* = 474.5 *Z* = 1.953 *p* = 0.05		*N _valid_* = 55 *T* = 412.0 *Z* = 3000 *p* = 0.003	
Bet/Gly/Suc/W0		Bet/Gly/Suc/W3		Bet/Gly/Suc/W6
	*N _valid_* = 49 *T* = 444.5 *Z* = 1.671 *p* = 0.095		*N _valid_* = 50 *T* = 356.0 *Z* = 2.717 *p* = 0.007	
Bet/Sor/W0		Beth/Sor/W3		Beth/Sor/W6
	*N _valid_* = 50 *T* = 556.0 *Z* = 0.787 *p* = 0.431		*N _valid_* = 52 *T* = 458.0 *Z* = 2.104 *p* = 0.035	
Pro/Gly/Sor/W0		Pro/Gly/Sor/W3		Pro/Gly/Sor/W6
	*N _valid_* = 49 *T* = 420.5 *Z* = 1.910 *p* = 0.06		*N _valid_* = 51 *T* = 292.0 *Z* = 3.478 *p* = 0.0005	
Gly/Glu/Sor/W0		Gly/Glu/Sor/W3		Gly/Glu/Sor/W6
	*N _valid_* = 50 *T* = 554.0 *Z* = 0.806 *p* = 0.420		*N _valid_* = 49 *T* = 315.0 *Z* = 2.959 *p* = 0.003	
Bet/EG0		Bet/EG3		Bet/EG6
	*N _valid_* = 48 *T* = 493.5 *Z* = 0.969 *p* = 0.332		*N _valid_* = 49 *T* = 398.5 *Z* = 2.129 *p* = 0.033	

## Data Availability

Not applicable.
